# Supplementary figures and images for: Cough dynamics in adults receiving tuberculosis treatment
Source: PLoS One. 2020 Jun 8;15(6):e0231167. doi: 10.1371/journal.pone.0231167 (PMC7279573; doi:10.1371/journal.pone.0231167)

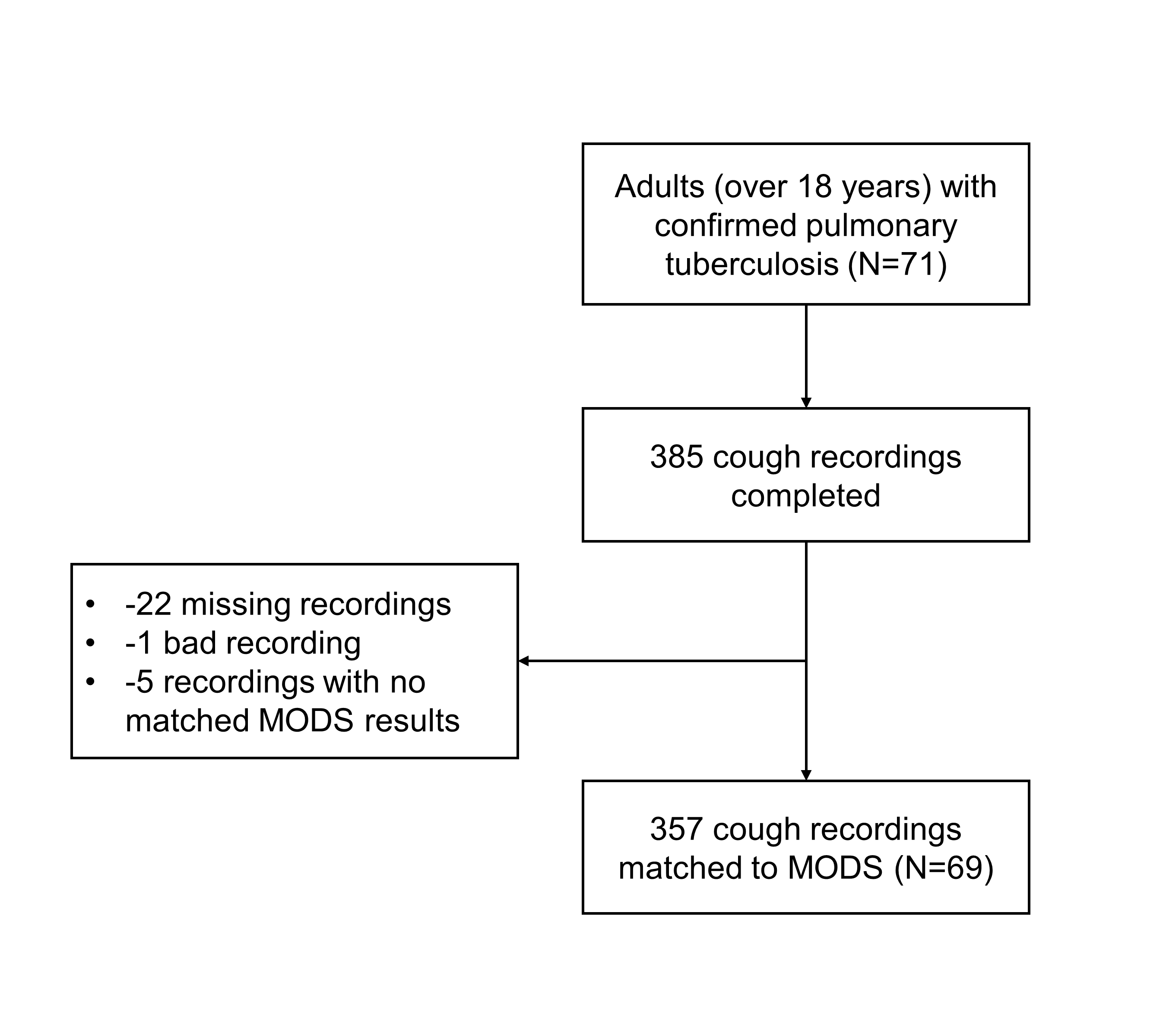

Supplement: S1 Fig — Shown here are the total number of cough recordings and microbiologic testing completed over the course of the study. 357 cough recordings (from 69 study participants) were matched to a microbiology result. (TIF) [file pone.0231167.s001.tif]

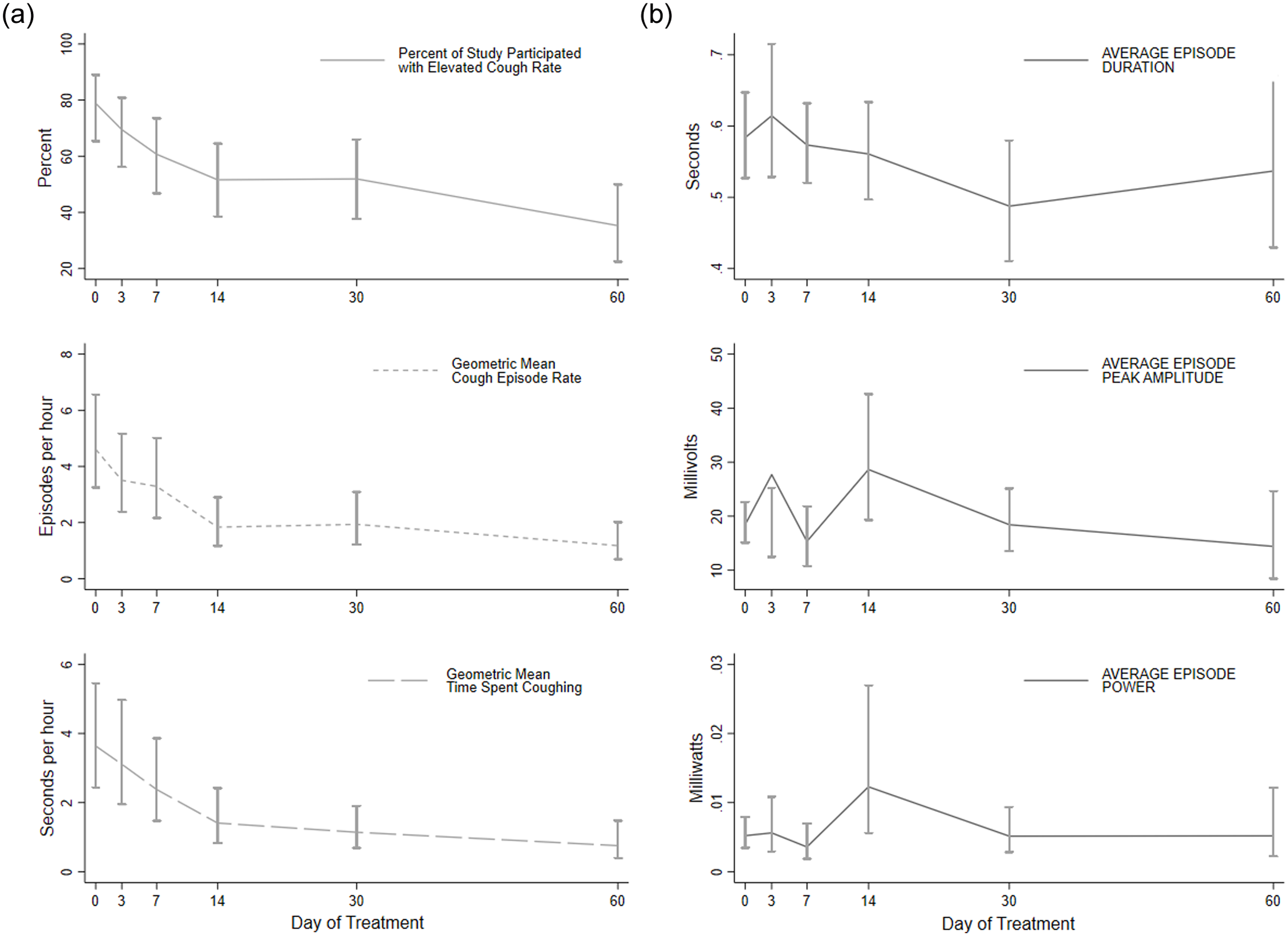

Supplement: S2 Fig — Shown here are the percentages of study participants with an elevated cough rate, and the geometric means of other characteristics of cough episodes, on specific days of treatment. By day 14 of treatment, 48% of patients had clinically normal cough rates. Recordings were taken as having occurred sufficiently near to the target day if they occurred strictly prior to the start or treatment, or on the first day of treatment (target day 0), or within +/-2 days (target day 3 and 7), +/- 7 days (target day 14), or +/-20 days (target days 30 and 60) of the target date. (TIF) [file pone.0231167.s002.tif]

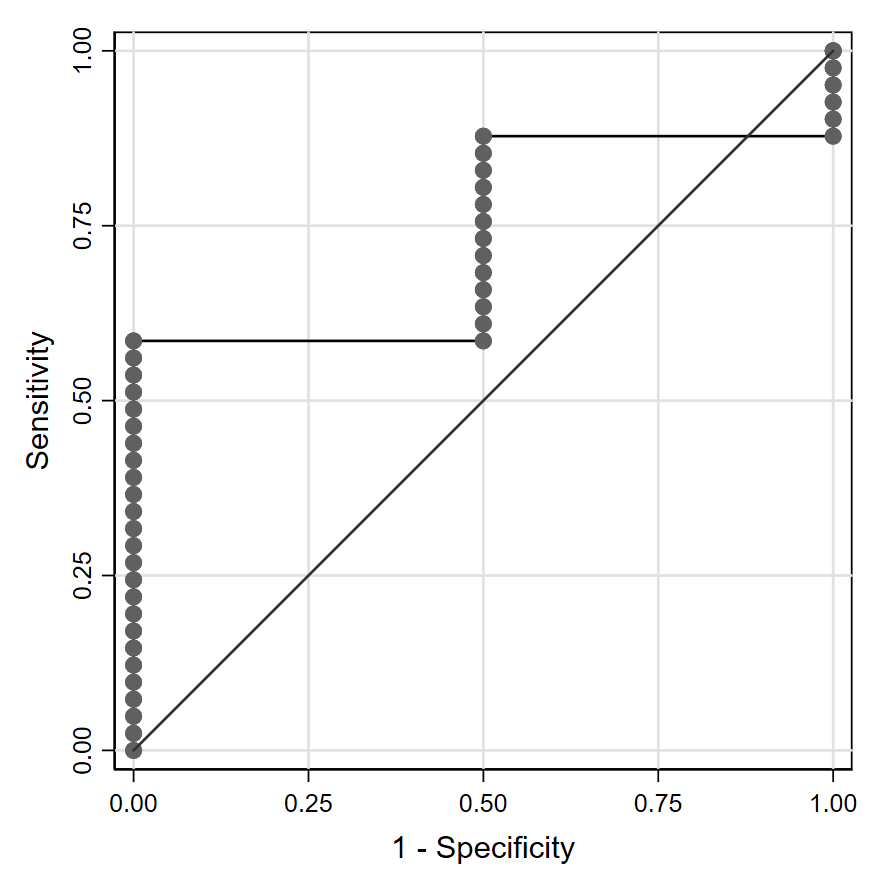

Supplement: S3 Fig — Shown here are receiver-operator curves (ROC) for MODS positivity versus TOTAL TIME COUGHING in 52 pre-treatment recordings (47 MODS positive and 5 MODS negative). AUC = 0.73; Best cut-off = 1.9 episodes/hour; Sensitivity = 85%; Specificity = 50%. (TIF) [file pone.0231167.s003.tif]
