# Supplementary material for: Cough dynamics in adults receiving tuberculosis treatment
Source: PLoS One. 2020 Jun 8;15(6):e0231167. doi: 10.1371/journal.pone.0231167 (PMC7279573; doi:10.1371/journal.pone.0231167)
Supplement: S1 Table — Shown here is a heat map representing non-parametric correlation coefficients between cough features, based on 43 of 52 pre-treatment cough recordings where at least one cough was recorded during the 4-hour recording. Results on treatment days 3–60 are similar (not shown). (DOCX) [file pone.0231167.s004.docx]

**S1 Table. Spearman Correlation Between Cough Features.**

|  | **AVERAGE EPISODE DURATION (seconds** | **AVERAGE EPISODE PEAK AMPLITUDE (millivolts)** | **AVERAGE EPISODE POWER (milliwatts)** | **TOTAL TIME COUGHING (seconds/hour)** | **TOTAL POWER EXPENDED COUGHING (milliwatts/hour)** |
| --- | --- | --- | --- | --- | --- |
| **AVERAGE EPISODE DURATION (seconds)** |  |  |  |  |  |
| **AVERAGE EPISODE PEAK AMPLITUDE (millivolts)** | 0.132 |  |  |  |  |
| **AVERAGE EPISODE POWER (milliwatts)** | 0.224 | 0.872 |  |  |  |
| **TOTAL TIME COUGHING (seconds/hour)** | 0.333 | 0.081 | 0.134 |  |  |
| **TOTAL POWER EXPENDED COUGHING (milliwatts/hour)** | 0.293 | 0.409 | 0.579 | 0.660 |  |
| **COUGH EPISODE FREQUENCY (episodes/hour)** | 0.198 | 0.020 | 0.067 | 0.961 | 0.660 |

| **Spearman correlation:** | |
| --- | --- |
|  | 0.80-1.00 |
|  |  |
|  | 0.60-0.79 |
|  |  |
|  | 0.40-0.59 |
|  |  |
|  | 0.20-0.39 |
|  |  |
|  | 0.00-0.19 |

Shown here is a heat map representing non-parametric correlation coefficients between cough features, based on 43 of 52 pre-treatment cough recordings where at least one cough was recorded during the 4-hour recording. Results on treatment days 3-60 are similar (not shown).
