# Supplementary material for: Cough dynamics in adults receiving tuberculosis treatment
Source: PLoS One. 2020 Jun 8;15(6):e0231167. doi: 10.1371/journal.pone.0231167 (PMC7279573; doi:10.1371/journal.pone.0231167)
Supplement: S2 Table — Shown here are intraclass correlation coefficients for each cough feature, describing the proportion of variability in each feature explained by within-individual variability. For example, 29% of the variability in COUGH EPISODE FREQUENCY can be explained by within-individual variability. (DOCX) [file pone.0231167.s005.docx]

**S2 Table. Intraclass Correlation Coefficients.**

|  | **AVERAGE EPISODE DURATION (seconds)** | **AVERAGE EPISODE PEAK AMPLITUDE**  **(millivolts)** | **AVERAGE EPISODE POWER (milliwatts)** | **TOTAL TIME COUGHING**  **(seconds/hour)** | **TOTAL POWER EXPENDED COUGHING (milliwatts/hour)** | **COUGH EPISODE FREQUENCY (episodes/hour)** |
| --- | --- | --- | --- | --- | --- | --- |
| **ICC** | 0.10  (0.03, 0.29) | 0.13  (0.05, 0.33) | 0.20  (0.09, 0.37) | 0.23  (0.12, 0.40) | 0.27  (0.15, 0.44) | 0.29  (0.17, 0.46) |

Shown here are intraclass correlation coefficients for each cough feature, describing the proportion of variability in each feature explained by within-individual variability. For example, 29% of the variability in COUGH EPISODE FREQUENCY can be explained by within-individual variability.
