# Supplementary material for: Cough dynamics in adults receiving tuberculosis treatment
Source: PLoS One. 2020 Jun 8;15(6):e0231167. doi: 10.1371/journal.pone.0231167 (PMC7279573; doi:10.1371/journal.pone.0231167)
Supplement: S3 Table — a-c. Association Between Cough Features and Microbiological Outcomes. These tables examine the extent to which specific features were predictive of microbiological outcomes. The primary microbiological outcome of interest was MODS time to positivity (TTP). Secondarily, we also considered the microbiological outcomes of MODS positivity (+/-) and smear positivity (+/-). All cough features are log-transformed (natural log AVERAGE EPISODE DURATION, natural log AVERAGE EPISODE PEAK AMPLITUDE, etc.). All models are bivariable models, unadjusted for treatment day or other factors, as it is expected that the relationship between characteristics of cough and treatment day was explained by microbiological response. All models include only recordings with at least one recorded cough episode included (complete case analysis). TTP models were Tobit models to account for the structure of the TTP data (TTP results 0 and 21 are treated as continuous, and TTP results of 22 or greater (equivalent to a negative MODS culture) are right-censored. MODS (+/-) and smear models were logistic models. All models included a random effect to account for within-patient variability. Log-likelihood (LL) and Akaike’s information criterion (AIC) were compared between models with each feature as the independent variable, where the model with the lowest AIC suggests that this feature is the strongest individual predictor of smear positivity. (DOCX) [file pone.0231167.s006.docx]

**S3 Tables a-c. Association Between Cough Features and Microbiological Outcomes.**

These tables examine the extent to which specific features were predictive of microbiological outcomes. The primary microbiological outcome of interest was MODS time to positivity (TTP). Secondarily, we also considered the microbiological outcomes of MODS positivity (+/-) and smear positivity (+/-). All cough features are log-transformed (natural log AVERAGE EPISODE DURATION, natural log AVERAGE EPISODE PEAK AMPLITUDE, etc.). All models are bivariable models, unadjusted for treatment day or other factors, as it is expected that the relationship between characteristics of cough and treatment day was explained by microbiological response. All models include only recordings with at least one recorded cough episode included (complete case analysis). TTP models were Tobit models to account for the structure of the TTP data (TTP results 0 and 21 are treated as continuous, and TTP results of 22 or greater (equivalent to a negative MODS culture) are right-censored. MODS (+/-) and smear models were logistic models. All models included a random effect to account for within-patient variability. Log-likelihood (LL) and Akaike’s information criterion (AIC) were compared between models with each feature as the independent variable, where the model with the lowest AIC suggests that this feature is the strongest individual predictor of smear positivity.

**S3 Table a. Time to positive (TTP).**

| **TTP** |  | **Only recordings with at least one cough**  **(N=239)** | | |
| --- | --- | --- | --- | --- |
|  | **Beta**  **Coefficient** | **Log-Likelihood** | **Degrees of freedom** | **AIC** |
| **AVERAGE EPISODE DURATION (seconds)** | -3.77 (-6.52, -1.01)  (p=0.007) | -598.27 | 4 | 1205 |
| **AVERAGE EPISODE PEAK AMPLITUDE (millivolts)** | 0.52 (-0.57, 1.61)  (p=0.348) | -601.73 | 4 | 1211 |
| **AVERAGE EPISODE POWER (milliwatts)** | 0.30 (-0.29, 0.90)  (p=0.319) | -601.25 | 4 | 1211 |
| **TOTAL TIME COUGHING (seconds per hour)** | -1.43 (-2.16, -0.71)  (p<0.001) | -594.29 | 4 | 1197 |
| **TOTAL POWER EXPENDED COUGHING (milliwatts per hour)** | -0.37 (-0.80, 0.06)  (p=0.089) | -600.33 | 4 | 1209 |
| **COUGH EPISODE FREQUENCY (episodes per hour)** | -1.55 (-2.43, -0.67)  (p=0.001) | -595.79 | 4 | 1200 |

Shown here is the beta coefficient describing time to positivity of the MODS result per one log increase in cough feature. For example, a 1-log increase in cough episode frequency (episodes per hour) is associated with decrease in time to positivity of 1.6 days.

**S3 Table b: MODS positivity.**

| **MODS** |  | **Only recordings with at least one cough**  **(N=239)** | | |
| --- | --- | --- | --- | --- |
|  | **Odds Ratio** | **Log-Likelihood** | **Degrees of freedom** | **AIC** |
| **AVERAGE EPISODE DURATION (seconds)** | 2.20 (1.08, 4.51)  (p=0.030) | -156.58 | 2 | 319 |
| **AVERAGE EPISODE PEAK AMPLITUDE (millivolts)** | 0.84 (0.64, 1.09)  (p=0.192) | -158.23 | 2 | 323 |
| **AVERAGE EPISODE POWER (milliwatts)** | 0.90 (0.78, 1.04)  (p=0.145) | -158.01 | 2 | 322 |
| **TOTAL TIME COUGHING (seconds per hour)** | 1.32 (1.09, 1.61)  (p=0.005) | -154.61 | 2 | 315 |
| **TOTAL POWER EXPENDED COUGHING (milliwatts per hour)** | 1.05 (0.95, 1.16)  (p=0.346) | -158.67 | 2 | 323 |
| **COUGH EPISODE FREQUENCY (episodes per hour)** | 1.34 (1.07, 1.68)  (p=0.012) | -155.59 | 2 | 317 |

Shown here are odds ratios (OR) describing the odds of a positive MODS result per one log increase in cough feature. For example, a 1-log increase in cough episode frequency (episodes per hour) is associated with a 34% increase in the odds of a positive MODS result.

**S3 Table c. Smear positivity.**

|  |  | **Only recordings with at least one cough (N=239)** | | |
| --- | --- | --- | --- | --- |
|  | **Odds Ratio** | **Log-Likelihood** | **Degrees of freedom** | **AIC** |
| **AVERAGE EPISODE DURATION (seconds)** | 2.38 (1.13, 5.00)  (p=0.023) | -159.86 | 3 | 326 |
| **AVERAGE EPISODE PEAK AMPLITUDE (millivolts)** | 0.88 (0.68, 1.15)  (p=0.347) | -162.29 | 3 | 331 |
| **AVERAGE EPISODE POWER (milliwatts)** | 0.94 (0.81, 1.08)  (p=0.364) | -162.32 | 3 | 331 |
| **TOTAL TIME COUGHING (seconds per hour)** | 1.55 (1.25, 1.93)  (p<0.001) | -152.81 | 3 | 312 |
| **TOTAL POWER EXPENDED COUGHING (milliwatts per hour)** | 1.12 (1.01, 1.25)  (p=0.037) | -160.43 | 3 | 326 |
| **COUGH EPISODE FREQUENCY (episodes per hour)** | 1.64 (1.27, 2.12)  (p<0.001) | -153.47 | 3 | 313 |

Shown here are odds ratios (OR) describing the odds of a positive smear result per one log increase in cough feature. For example, a 1-log increase in cough episode frequency (episodes per hour) is associated with a 64% increase in the odds of a positive smear result.
