# Supplementary material for: Cough dynamics in adults receiving tuberculosis treatment
Source: PLoS One. 2020 Jun 8;15(6):e0231167. doi: 10.1371/journal.pone.0231167 (PMC7279573; doi:10.1371/journal.pone.0231167)
Supplement: Attachment — Submitted filename: PLOS PONE-D-19-31712.docx [file pone.0231167.s007.docx]

**Manuscript #**: PONE-D-19-31712

**Title:** COUGH DYNAMICS IN ADULTS RECEIVING TUBERCULOSIS

TREATMENT

**Review:**

The study is interesting, but the total number of subjects included in the study (n=69) is very small to reach any substantial conclusion. These numbers further reduced significantly in the sub groups, the researchers have chosen i.e. (Drug resistant n=8; HIV positive n=8; Diabetics n=6; smokers n=13) etc.

Hence, with such a small group of participants, the hypothesis, which the authors are putting forward, cannot be substantiated.

A higher numbers of subjects are required to be chosen enabling the authors to establish a statistical significance of the study.
